# Association between pulmonary hemorrhage and CPAP failure in very preterm infants

**DOI:** 10.3389/fped.2022.938431

**Published:** 2022-09-09

**Authors:** Li Wang, Li-li Zhao, Jia-ju Xu, Yong-hui Yu, Zhong-liang Li, Feng-juan Zhang, Hui-min Wen, Hai-huan Wu, Li-ping Deng, Hui-yu Yang, Li Li, Lan-lan Ding, Xiao-kang Wang, Cheng-yuan Zhang, Hui Wang

**Affiliations:** ^1^The First Affiliated Hospital of Sun Yat-sen University, Guangzhou, China; ^2^Shandong Provincial Hospital Affiliated to Shandong First Medical University, Jinan, China; ^3^Liaocheng People's Hospital, Liaocheng, China; ^4^Yantai Yuhuangding Hospital, Yantai, China; ^5^W.F. Maternal and Child Health Hospital, Weifang, China; ^6^The First Affiliated Hospital of Shandong First Medical University, Jinan, China; ^7^Hebei PetroChina Central Hospital, Langfang, China; ^8^Baogang Third Hospital of Hongci Group, Baotou, China; ^9^Heze Municipal Hospital, Heze, China; ^10^Women and Children's Healthcare Hospital of Linyi, Linyi, China; ^11^Linyi People's Hospital, Linyi, China; ^12^Jinan Maternity and Child Health Care Hospital, Jinan, China; ^13^Central Hospital of Shandong Provincial Hospital Affiliated to Shandong First Medical University, Jinan, China

**Keywords:** very low birth weight infant, pulmonary hemorrhage, CPAP failure, multicenter study, very preterm infant

## Abstract

**Background:**

Pulmonary hemorrhage (PH) in neonates is a life-threatening respiratory complication. We aimed to analyze the perinatal risk factors and morbidity with PH among very preterm infants in a large multicenter study.

**Methods:**

This was a multicenter case–control study based on a prospective cohort. Participants included 3,680 in-born infants with a gestational age at 24–32 weeks (birth weight <1,500 g) who were admitted between January 1, 2019, and October 31, 2021. All infants were divided into two groups, namely, the PH and no-PH groups, at a ratio of 1:2 according to the following factors: gestational age (GA), birth weight (BW), and the Score for Neonatal Acute Physiology with Perinatal extension II (SNAPPE II). Perinatal factors and outcomes were compared between the two groups by logistic regression analyses.

**Results:**

A total of 3,680 infants were included in the study, and the number of identified cases of PH was 262 (7.1%). The incidence was 16.9% (136/806) for neonates with extremely low BW (BW < 1,000 g) infants. The multivariate analysis showed that CPAP failure (OR 2.83, 95% CI 1.57, 5.08) was significantly associated with PH. PH was associated with a high likelihood of death (OR 3.81, 95% CI 2.67, 5.43) and bronchopulmonary dysplasia (BPD) (≥grade II) (OR 1.58, 95% CI 1.00, 2.48).

**Conclusions:**

In this multicenter case–control study based on a prospective cohort, PH to be common among VLBW infants. PH is associated with significant morbidity and mortality, and perinatal management, especially CPAP failure. Respiratory management strategies to decrease the risk of PH should be optimized.

## Introduction

Pulmonary hemorrhage (PH) in newborns is an adverse event leading to critical complications and even death (1), especially in very low birth weight (VLBW) infants who required invasive ventilation and intensive care after birth. PH occurs in 4-12% (2, 3, 5–7) of VLBW infants and is associated with significant morbidity and mortality (8–10). Ahmad et al. (10) reported that the incidence of PH is highest in neonates born at 24 weeks gestation, and this incidence gradually decreases in those born up to 32 weeks gestation. The incidence of pulmonary hemorrhage in infants born after 32 weeks of gestational age remained around baseline.

The risk factors for PH, such as smaller gestational age (GA), lower birth weight (BW), surfactant use, patent ductus arteriosus (PDA) and coagulopathy, have been described in previous studies (2–4). A prospective study found that continuous positive airway pressure (CPAP) failure was closely associated with PH (11). In recent years, with improvements in living conditions and the development of medical technology, the proportion of extremely premature infants (GA <28 weeks) entering neonatal intensive care units (NICUs) in our country has gradually increased (12, 13). However, is PH still associated with previously identified perinatal risk factors in the current population? We conducted a large-sample study to analyze the perinatal risk factors and morbidity with PH among very preterm infants.

## Methods

### Study design and population

We carried out a multicenter case–control study based on a prospective cohort from the Sino-northern Neonatal Network (SNN), a large, multicenter data set. The cohort study included all infants with a gestational age at 24–32 weeks (birth weight < 1,500 g) who were admitted between January 1, 2019, and October 31, 2021. Infants who were out-born, who had redirection of intensive care (14) and who had congenital malformations were excluded. We utilized this population for baseline data analysis. To remove confounding factors, we performed 1:2 matching using this cohort.

### Data collection

The SNN has been implemented since January 1, 2018. The SNN includes teaching hospitals and non-teaching hospitals. The database collects prenatal information and neonatal treatment and complications during hospitalization, and the data were collected by trained medical staff using a standardized operating procedure (15, 16).

### Study variables

PH was defined as a considerable amount of bloody fluid suctioned from the endotracheal tube concomitantly followed by acute clinical deterioration and the presentation of fluffy or ground-glass opacities throughout the lung fields on chest X-ray. That is, it met the diagnostic criteria for PH (International Classification of Disease Revision 9 code 770.3). All perinatal variables occurred before PH. CPAP failure was defined as follows: CPAP failure within the first 72 h of life, intubation, and the initiation of mechanical ventilation (MV) (17, 18). Hypothermia was defined as an axillary temperature of <36.5°C, according to the WHO (19). Cold stress or mild hypothermia was defined as a temperature 36.0–36.4°C, moderate hypothermia was defined as a temperature 32.0–35.9°C, and severe hypothermia was defined as a temperature below 32°C. Early-onset neonatal sepsis (EOS) was defined as premature rupture of membranes more than 18 h before delivery, infectious disease development within 72 h after birth and abnormal values for 2 or more non-specific infection indications: WBC < 5 × 10^9^/L or WBC > 20 × 10^9^/L; C-reactive protein (CRP) ≥10 mg/L; platelet (PLT) ≤ 100× 10^9^/L; and procalcitonin (PCT) > 2 ng/ml. If blood or cerebrospinal fluid culture was positive, then culture-positive septicaemia was diagnosed (20, 21). Bronchopulmonary dysplasia (BPD) was defined as the requirement to increase the inspired fraction oxygen at the corrected gestational age of 36 weeks (22).

### Statistical analysis

**Figure 1:**
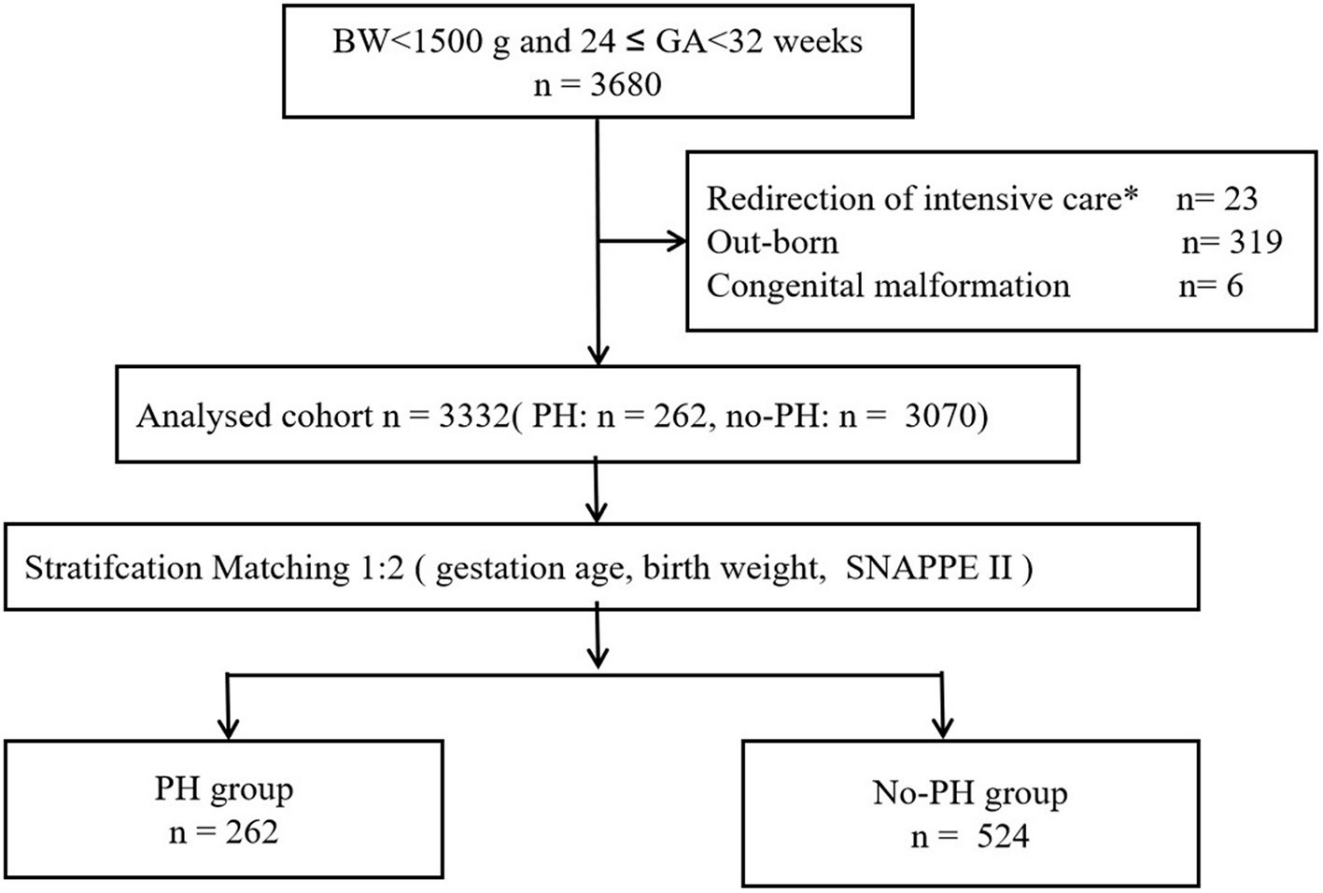
Flow diagram of the study population.

The data are expressed using medians [M (*Q*_1_, *Q*_3_)] or percentages. We performed univariate analysis using the Kruskal–Wallis test or chi-square test. The multivariate logistic regression was further used to evaluate the odds ratios (ORs), with adjustment for perinatal factors. Neonates weighing <1,500 g who were born between 24 and 32 weeks gestation were included in this study (Figure 1). The baseline demographic characteristics are presented in Table 1 (*n* = 3, 332). The participants were divided into two groups (PH and no-PH groups) at a ratio of 1:2 according to three factors: GA (±1 week), birth weight (±200 g) and Score for Neonatal Acute Physiology with Perinatal extension II SNAPPE II (±10). After 1:2 matching, 524 infants in the control group were included for further analyses (Figure 1). A *P*-value < 0.05 was considered statistically significant. SPSS v. 25.0 (SPSS Inc., Chicago, Illinois) was used for statistical analysis.

**Table 1:** Characteristics of VLBW infants with and without PH.

	**PH (*n =* 262)**	**No-PH (*n =* 3,070)**	P^*^
GA (weeks)	28 (26, 29)	29 (28, 31)	<0.001
BW (g)	980 (850, 1,152)	1,200 (1,022, 1,350)	<0.001
SGA	40 (15.3)	405 (13.2)	0.316
Sex (boy)	133 (50.8)	1,618 (52.7)	0.638
Maternal hypertension	78 (29.8)	963 (31.4)	0.653
GDM	42 (16.0)	512 (16.7)	0.831
Antenatal magnesium sulfate use	132 (50.4)	1,595 (51.9)	0.721
Antenatal steroid use	190 (72.5)	2,222 (72.4)	0.796
SNAPPE - II	30 (17, 48)	19 (10, 32)	<0.001

Data are presented as the median (Q1, Q3) or n (%).

* Kruskal-Wallis or chi-square test.

GA, gestational age; BW, birth weight; SGA, small for gestational age; PROM, premature rupture of membranes; GDM, gestational diabetes mellitus.

## Results

A total of 3,680 infants were enrolled in the study on their day of birth, of which 262 infants (7.1%) were diagnosed with PH (Figure 1). Throughout the study period, the incidence of PH among extremely low birth weight (BW < 1000 g) infants was 16.9% (136/806). The final cohort had a median BW and GA of 1,180 (1,000–1,320) g and 29 (28–30) weeks, respectively.

In the matched sample, the univariate analyses indicated that the SpO2/FiO2 ratio, surfactant use, shock, CPAP failure and moderate/severe hypothermia were associated with PH (Table 2). There were no differences in the delivery room intubation rate, 1- and 5-min Apgar scores <7, initial CPAP support or initial MV support between infants with PH and controls (Table 2). After adjustment for risk factors, CPAP failure (OR 2.83, 95% CI 1.57, 5.08) remained significantly associated with PH (Table 3).

**Table 2:** Baseline characteristics after 1:2 matching in the PH and no-PH groups.

	**PH (*n =* 262)**	**No-PH (*n =* 524)**	P^*^
GA (weeks)	28 (26, 29)	28 (27, 29)	0.585
BW (g)	980 (850, 1,152)	1,000 (850, 1,177)	0.550
Sex (boy)	161 (61.4)	282 (53.8)	0.067
SGA	20 (7.6)	57 (10.8)	0.149
Maternal hypertension	84 (32.1)	168 (32.1)	1.000
GDM	41 (15.6)	93 (17.7)	0.461
Antenatal magnesium sulfate use	149 (56.9)	279 (53.2)	0.336
Antenatal steroid use	207 (79.0)	396 (75.6)	0.283
Incomplete course	126 (48.1)	236 (45.0)	
Completed course	81 (30.9)	160 (30.5)	
Delayed umbilical cord clamping	80 (30.5)	153 (29.2)	0.699
Delivery room intubation	117 (44.7)	267 (60.0)	0.096
Resuscitation (chest compression)	35 (13.4)	92 (17.6)	0.132
Apgar score at 1 min	6 (4, 8)	6 (4, 8)	0.881
Apgar score at 5 min	8 (6, 9)	8 (6, 9)	0.518
SNAPPE II	30 (17, 48)	29 (14, 47)	0.929
Shock	80 (30.5)	79 (17.0)	<0.001
Moderate/severe hypothermia	123 (46.9)	230 (43.9)	0.024
SpO_2_/FiO_2_	1.6 (1.0, 2.4)	2.4 (1.6, 3.3)	<0.001
Surfactant use	132 (50.5)	210 (40.1)	0.015
Dose of surfactant (mg/kg)	193.9 (100, 200)	210.0 (100, 200)	0.550
Initial MV	111 (42.4)	189 (36.1)	0.087
Initial CPAP	66 (25.2)	158 (30.2)	0.146
CPAP failure	33 (12.6)	22 (4.2)	<0.001
Initial systolic blood pressure instability	31 (11.8)	48 (9.20)	0.240
Moderate/large PDA	70 (26.7)	86 (16.4)	0.001
EOS	20 (7.6)	47 (9.0)	0.527

Data are presented as the median (Q1, Q3) or n (%).

* Kruskal-Wallis or chi-square test.

GA, gestational age; BW, birth weight; SGA, small for gestational age; PROM, premature rupture of membranes; GDM, gestational diabetes mellitus; CPAP, continuous positive airway pressure; PDA patent ductus arteriosus; EOS, early-onset neonatal sepsis.

**Table 3:** Risk factors for PH as determined by logistic regression.

	Adjusted OR^*^	**95% CI**	***P***
GA (weeks)	1.39	0.98, 1.59	0.124
BW (g)	1.00	0.99, 1.00	0.56
Sex (boy)	1.29	0.93, 1.78	0.134
SGA	1.14	0.66, 1.97	0.625
SNAPPE II	1.00	0.98, 1.01	0.464
Moderate/severe hypothermia	0.93	0.66, 1.31	0.691
CPAP failure	2.83	1.57, 5.08	0.001
SpO_2_/FiO_2_	0.86	0.64, 1.15	0.311
Surfactant use	1.27	0.95, 2.18	0.139
Shock	1.34	0.85, 2.15	0.210
Moderate/large PDA	1.55	1.06, 2.27	0.023

OR, odds ratio; CI, confidence interval; GA, gestational age; BW, birth weight; SGA, small for gestational age; CPAP, continuous positive airway pressure; PDA patent ductus arteriosus; SpO_2_/FiO_2_, the ratio of partial oxygen pressure to fraction of inspired oxygen.

* All significant variables in the univariate analysis.

The multivariate analysis showed that death and BPD (≥grade II) were significantly associated with PH (Table 4). Further study found that among the infants who survived, there was no significant difference in the length of hospital stay between those with PH and the controls (Table 4).

**Table 4:** Neonatal mortality and morbidities in the PH and no-PH groups in univariate and multivariate analyses.

	**PH (*n =* 262)**	**No-PH (*n =* 524)**	Adjusted OR^*^(95% CI)	***P***
Death	130 (49.6)	117 (22.3)	3.81 (2.67, 5.43)	<0.001
Moderate-to-severe BPD	55 (21.0)	76 (14.5)	1.58 (1.00, 2.48)	0.047
IVH (grades 3 or 4)	30 (11.5)	31 (5.9)	1.42 (0.79, 2.54)	0.240
NEC (Bell stage ≥ 2)	17 (6.5)	18 (3.4)	1.53 (0.71, 3.28)	0.276
Stage 3 or higher ROP	14 (5.3)	32 (6.1)	0.93 (0.45, 1.89)	0.839
Hospital stay^‡^	67 (47, 80)	58 (46, 73)	0.99 (0.98, 1.02)	0.999

OR, odds ratio; CI, confidence interval; BPD, bronchopulmonary dysplasia; IVH, intraventricular hemorrhage; NEC, necrotizing enterocolitis; ROP, retinopathy of prematurity; SpO_2_/FiO_2_, the ratio of partial oxygen pressure to fraction of inspired oxygen.

* Adjusted for GA, BW, SGA, Sex, and SNAPPE II.

‡ Survival to discharge.

## Discussion

This was a multicenter, case–control study based on a large prospective cohort conducted to investigate the association between perinatal factors and PH in China. In our study, the incidence of PH was 7.1% in VLBW infants, while it was as high as 16.9% in extremely low BW infants. Previous literatures found that the incidence of PH was 11–18.8% in extremely low BW infants (5, 13). Ahmad et al. (10) reported that the incidence of PH increased with decreasing gestational age. The overall mortality rate was 46.9% in infants with PH. The likelihood of overall death was 3.81-fold higher in infants with a diagnosis of PH than controls in this cohort. This result was similar to those in previous studies (2–4).

The multivariate analysis revealed an association of PH with CPAP failure but not with moderate/severe hypothermia. Neonatal cold injury syndrome has been shown to be a major risk factor for PH in low- and middle-income countries (23). When infants are in a state of severe hypothermia, pulmonary vascular tension decreases, resulting in increased pulmonary blood volume and pulmonary vascular bed dilation, leading to oedema and PH (24). Our previous study found that the incidence of hypothermia on admission (body temperature <36.5°C) in VLBW infants was 88.2% (12). With the implementation of a quality improvement program (17), the incidence of severe hypothermia in our cohort decreased gradually, which may also improve the rate of PH. The next step will focus on the relationship between severe hypothermia and PH.

Many studies have shown that PDA is associated with the occurrence of PH (2, 3). These results were similar to those in our study. PDA can cause a left-to-right shunt, resulting in high flow and a high-pressure state in the pulmonary vascular bed. Kluckow et al. (25) showed that PH was associated with significant catheter shunt and high pulmonary blood flow and that increased catheter diameter and right ventricular outflow may lead to PH.

We found that the characteristics of infants with PH included perinatal factors such as CPAP failure. This was consistent with our previous finding (2). In this study, we found no differences in initial CPAP respiratory support and initial MV respiratory support between the two groups. This may be attributed to the type of nasal interface used, use of a surfactant and the CPAP device (26), leading to the instability of positive end-expiratory pressure (PEEP). The need for ventilation with positive pressure and oxygen leads to excessive alveolar distension, causing stress damage to the alveolar capillaries, thereby contributing to the pathogenesis of PH (27). Piacentini et al. (28) reported that the application of a moderate level of PEEP had protective effect, reducing the formation of oedema and PH. In China, the number of doctors, the ratio of nurses to beds, the ratio of doctors to nurses, the ratio of doctors with graduate degrees, and the ratio of nurses with university or higher certificates are all insufficient, often making it difficult to provide high-quality care. These factors also support the need to strictly follow respiratory management guidelines (29) in neonates to avoid CPAP failure. Therefore, we should optimize perinatal management, especially respiratory management, to avoid PH in low- and middle-income countries. In a word, the stable time of delivery room should be extended until after birth, and the stable state should be achieved as soon as possible after entering the NICU to avoid excessive fluctuations of various indicators.

Our study demonstrated that surviving infants with PH had adverse outcomes, including a relatively high rate of moderate-to-severe BPD after correction for potential confounders; this finding is consistent with those of previous reports (30). Active management is critical when PH occurs. After stopping the hemorrhage, high-frequency oscillatory ventilation (HFOV) may be an option for the treatment of respiratory failure (31). However, a longer duration of respiratory support (4) and a higher PEEP (30) can cause inflammatory responses, resulting in BPD (32). Therefore, for surviving infants with PH, we should pay attention to postrespiratory management and reduce the incidence of severe BPD.

An important strength of our study was that it was a multicenter, case-control study based on a prospective cohort and a complete data set from 47 NICUs in China. The SNN is a prospective collection of cases, and all variables are collected in chronological order. In our study, we focused on the analysis of perinatal management after delivery, including admission temperature, respiratory support methods and other aspects, to effectively guide clinicians. In developing countries, with the rapid development of medical technology and equipment, medical staff should pay more attention to the details, optimization and refinement of perinatal management, especially initial respiratory stability. There were some limitations in our study. The limitations of this study included some missing clinical information. Multicenter NICUs may have different treatment levels and fail to provide homogeneous management. Based on the findings of this study, our next step is to conduct a quality improvement program to reduce the incidence of PH.

In conclusions, we demonstrated that PH increased the risks of mortality and BPD (≥grade II) and was associated with perinatal management, especially CPAP failure. Respiratory management strategies to decrease the risk of PH should be optimized. At the same time, the stable time of delivery room should be extended until after birth, and the stable state should be achieved as soon as possible after entering the NICU to avoid excessive fluctuations of various indicators.

## Data availability statement

The original contributions presented in the study are included in the article/supplementary material, further inquiries can be directed to the corresponding author.

## Ethics statement

The Institutional Review Board of Shandong Provincial Hospital Affiliated with Shandong University approved the project (Ethical Approval Number: LCYJ: No. 2019-132). Informed consent was signed by the legal guardian of all participants.

## Author contributions

Y-hY is responsible for the design and review of the manuscript. LW played a role in the analysis and interpretation of the data and in preparing and drafting the manuscript. L-lZ, J-jX, Y-hY, Z-lL, F-jZ, H-mW, H-hW, L-pD, H-yY, LL, L-lD, and X-kW designed the data collection instruments, collected data, carried out the initial analyses, and reviewed and revised the manuscript. C-yZ and HW conceptualized and designed the study, coordinated and supervised data collection, and critically reviewed the manuscript for important intellectual content. All authors have read and approved the manuscript.

## Funding

This study was supported by the Shandong Medical Association Clinical Research Fund- Qilu Special Project (YXH2022DZX0200X).

## Conflict of interest

The authors declare that the research was conducted in the absence of any commercial or financial relationships that could be construed as a potential conflict of interest.

## Publisher's note

All claims expressed in this article are solely those of the authors and do not necessarily represent those of their affiliated organizations, or those of the publisher, the editors and the reviewers. Any product that may be evaluated in this article, or claim that may be made by its manufacturer, is not guaranteed or endorsed by the publisher.
